# Myoclonus following a Peripheral Nerve Block

**DOI:** 10.1155/2013/213472

**Published:** 2013-07-11

**Authors:** Arlene J. Hudson, Kevin B. Guthmiller, Marian N. Hyatt

**Affiliations:** ^1^Department of Anesthesiology, Uniformed Services University of the Health Sciences, 4301 Jones Bridge Road, Bethesda, MD 20814, USA; ^2^San Antonio Military Medical Center, 3551 Roger Brooke Drive, San Antonio, TX 78234m, USA

## Abstract

Myoclonus is an extremely rare perioperative complication following neuraxial anesthesia. It has also been reported to occur due to peripheral nerve lesions. We report a case of self-limiting myoclonus following a routine peripheral nerve block in an otherwise healthy patient.

## 1. Introduction 

A 29-year-old otherwise healthy man presented for an elective left anterior cruciate ligament revision to be performed under a peripheral nerve block. Surgical anesthesia was accomplished with the Labat approach to the sciatic nerve and a live ultrasound guided femoral nerve block. Nerve stimulation was achieved for both nerve blocks at 0.4 mA. On the morning of postoperative day one, the patient developed an involuntary, painless, rhythmic movement of his left lower extremity consistent with spinal myoclonus.

Peripheral nerve blocks are routinely performed for ambulatory orthopedic procedures. Various techniques have been successfully employed including the use of peripheral nerve stimulators as well as ultrasound with excellent efficacy and patient safety. We report a case of myoclonus following a routine peripheral nerve block in an otherwise healthy patient. While myoclonus following neuraxial anesthesia is a rare but recognized phenomenon, myoclonus following a peripheral nerve block has not previously been described.

## 2. Case Report

A 29-year-old, 90 kilogram, Caucasian man presented for a left anterior cruciate ligament revision. He had previously undergone four uncomplicated left knee procedures all with neuraxial anesthesia. The patient gave consent for regional anesthesia and monitored anesthesia care. 

Two blocks for the left lower extremity were performed in a dedicated block area. Oxygen by face mask and routine monitors were applied. Sedation was provided with midazolam (2 mg IV) and fentanyl (100 mcg IV), and the patient remained alert and responsive throughout the procedure. A posterior approach to the sciatic nerve employing Labat's technique was performed. A nerve stimulator was used, and stimulation of the common peroneal segment of the sciatic nerve was achieved at 0.40 mA. Following a positive Raj sign, 30 mL of local anesthetic was injected in a routine fashion using 5 mL aliquots (10 mL of 1.5% mepivacaine with 1 : 400,000 epinephrine, plus 20 mL of 0.5% bupivacaine with 1 : 400,000 epinephrine). The patient was repositioned to the supine, and a femoral block was placed with a technique employing simultaneous live ultrasound (SonoSite MicroMaxx, SonoSite Inc., Bothell, WA; Linear 6–13 MHz) and nerve stimulation. The femoral nerve stimulation was achieved at 0.40 mA. Again, 30 mL of local anesthetic was injected using 5 mL aliquots (0.5% bupivacaine with 1 : 400,000 epinephrine.) 

The peripheral nerve blocks provided surgical anesthesia supplemented with dexmedetomidine and fentanyl sedation. The procedure was completed in 3 hours and 51 minutes with a tourniquet time of 125 minutes. The patient recovered overnight in the hospital and received two Percocet tablets (acetaminophen 325 mg/oxycodone 5 mg) and ketorolac (30 mg IV) for analgesia supplementation. On the morning of postoperative day 1, the patient developed an involuntary, painless, rhythmic movement of his left lower extremity. The patient's left ankle was observed to invert and remain inverted for approximately 1 second before returning to the neutral position. The rhythmic movement exhibited a frequency of 1 every 3 seconds and persisted during sleep as noted by the patient's spouse (see digital video, Supplemental Digital Content 1 in Supplementary Material available online at http://dx.doi.org/10.1155/2013/213472, which is a video of the patient's myoclonus). On exam, full strength and motor function were intact in all muscle groups with the involuntary rhythmic movement being absent during the voluntary motor movement. Residual numbness in the distribution of the femoral nerve was evident, but full sensation was present in the sciatic distribution. Vibratory and position senses were not tested, and cerebellar function was not assessed. A Babinski sign revealed down-going toes bilaterally.

The patient was discharged that morning with instruction for followup with orthopedics as well as the Acute Pain Service. Information pertaining to options for diagnosis was presented, and no immediate consultation was desired by the patient. According to a telephone conversation with the patient, the involuntary rhythmic movement persisted for a total of approximately 6–10 hours followed by spontaneous resolution. No further sequelae were noted. 

The authors have obtained written consent from the patient to publish this case report. 

## 3. Discussion

Myoclonus is any brief, involuntary twitching or movement of a muscle or a group of muscles. It is usually caused by muscle contractions (positive myoclonus), but myoclonus also can result from brief lapses of contraction (negative myoclonus). A variety of classification schemes have been developed to categorize myoclonic movements. The “neurophysiologic” classification is a commonly used scheme based on the neuroanatomical origin of electrical discharge and includes the categories cortical, subcortical, spinal, or peripheral myoclonus [1]. In most types of myoclonus, the pathophysiology is unclear although structural lesions, trauma, and abnormalities in neurotransmitter receptors are often present [2]. The neuroanatomical origin of abnormal discharge determines the expressed myoclonic pattern. This may be useful in determining the origin of clinical myoclonus. 

Cortical myoclonus arises from the sensorimotor cortex. The transmission of aberrant activity down the corticospinal pathway is characterized by arrhythmic jerks [3]. Subcortical myoclonus originates between the cortex and the spinal cord in the area of the thalamus or brainstem. Subcortical myoclonus is characterized by generalized jerks of the proximal limbs and axial muscles [3]. 

Spinal myoclonus is typically associated with a lesion in the spinal cord which may cause changes in the afferent signaling from peripheral and supraspinal structures. The duration of the involuntary movements is often longer and more variable than what is seen in cortical or subcortical myoclonus. The movements may be unilateral or bilateral and originate from a spontaneous motor neuron discharge in a single segment of the spinal cord. In one type of spinal myoclonus called segmental spinal myoclonus, the discharge is limited to the level of the lesion and movement is characterized by nonstimulus sensitive rhythmic (0.5–3 Hz) movement. A second type of spinal myoclonus, propriospinal myoclonus, is typified by the propagation of myoclonic jerks along multiple levels of the cord with the myoclonic generator often at the thoracic level. The rhythmic movements are typically slow, bilateral, and usually more extensive than those seen with segmental spinal myoclonus [4, 5]. 

Lastly, peripheral myoclonus is hypothesized to be caused by lesions of the peripheral nerves that may alter sensory input and induce central reorganization [6]. Peripheral myoclonus is typically not stimulus sensitive and usually results in arrhythmic rapid (200–400 millisecond) jerks. The most commonly encountered peripheral myoclonus is hemifacial spasm. 

Our patient exhibited clinical signs suggestive of both spinal and peripheral myoclonus; however, the rhythmic, nonstimulus nature and frequency (0.3–0.5 Hz) of his foot inversion most closely approximates segmental spinal myoclonus. Unfortunately, our patient declined electrophysiological testing, and therefore, our diagnosis is not definitive. Although neuraxial anesthesia has been reported in the literature to cause spinal myoclonus, [7] to our knowledge there are no previous accounts of spinal myoclonus following a regional peripheral nerve block. 

Various mechanisms have been suggested as the cause of spinal myoclonus. In general, the loss of inhibitory function of local dorsal horn interneurons, abnormal hyperactivity of local anterior horn cells, and aberrant local axons reexcitation and loss of inhibition from suprasegmental descending pathways have all been suggested as possible etiologies [8]. Given the association of transient spinal myoclonus following neuraxial anesthesia, our patients may have experienced a spread of local anesthetic from the site of injection at the sciatic nerve into the dural sleeves of the nerve roots with migration into the subarachnoid space. This etiology would then be similar to that associated with neuraxial anesthesia and segmental spinal myoclonus. Given the lack of signs suggestive of spinal anesthesia, this explanation appears unlikely.

Peripheral nerve trauma may induce altered sensory inputs and disinhibition of anterior horn neurons via a local sensorimotor integration process. In fact, several cases of spinal myoclonus have been reported in the literature following injury to a peripheral nerve, and some cases have been successfully treated with injections of local anesthetic and botulism toxin. The ensuing success has been attributed to the interruption of abnormal afferent impulse generation [9]. Savrun et al. describe the onset of segmental spinal myoclonus following an ulnar nerve injury [10]. Involuntary movements emerged and intensified over a period of months eventually involving the patient's entire arm. Electromyography confirmed progression to the C5 and C8 nerve roots [10]. Likewise, Assal et al. reported a movement disorder following injury to the cutaneous branch of the deep peroneal nerve with subsequent myoclonus of the first dorsal interosseus muscle [6]. Like our patient, the movements were rhythmic and nonpainful. Proximal local anesthetic injection of the cutaneous branch of the deep peroneal nerve suppressed the myoclonus, suggesting a peripheral origin of the myoclonus with a relay within the spinal cord likely at the L5-S1 level. Shin et al. reported a patient with a known traumatic lesion to the femoral nerve who experienced temporary resolution of the myoclonus with lumbar spinal anesthesia as well as local anesthetic block of the femoral nerve [11], again suggesting a peripheral origin with spinal relay involvement. In all of these cases, however, the movement disorder progressed over a period of months following a permanent peripheral nerve injury. Moreover, other than transient resolution with local anesthetic, in all of these cases the myoclonus persisted. 

Ankle inversion, as exhibited by our patient, is accomplished by muscles innervated by branches of the sciatic nerve, specifically the posterior tibial and anterior tibial nerves. The posterior tibial nerve is a branch of the tibial nerve and is comprised of fibers from the L4-5 nerve roots [12]. It innervates muscles which act to invert the ankle while it is in plantarflexion [13]. The anterior tibial nerve is a branch of the deep peroneal nerve and is comprised of fibers from the L5 nerve root. It supplies muscles in the anterior compartment that act to invert the foot while it is in dorsiflexion [12]. Thus, the act of ankle inversion can be accomplished both via anterior or posterior tibial stimulation. Our patient did not appear to be in either excessive plantar or dorsiflexion, and upon examination it was difficult to determine whether the anterior or posterior muscle groups were the primary effectors of ankle movement. 

Various techniques for peripheral nerve blocks have been successfully employed including the use of peripheral nerve stimulators as well as ultrasound with excellent efficacy and patient safety. Despite the caution taken with the placement of the two nerve blocks, it is reasonable to consider direct injury or toxicity to the sciatic nerve as the cause of our patient's transient myoclonus. If indeed the sciatic nerve or its branches sustained reversible injury during the regional block, abnormal afferent input generated at the lesion site could have altered the action of local inhibitory spinal interneurons and created a transient involuntary movement disorder. 

While myoclonus following neuraxial anesthesia is a recognized phenomenon, albeit exceedingly rare, to our knowledge this case is the first reported myoclonus following a peripheral nerve block.

## Supplementary Material

The supplementary digital file contains a short video of the patient's myoclonus on postoperative day one.
